# Mpox in Children and Adolescents during Multicountry Outbreak, 2022–2023

**DOI:** 10.3201/eid2910.230516

**Published:** 2023-10

**Authors:** Ana Hoxha, Steven M. Kerr, Henry Laurenson-Schafer, Nikola Sklenovská, Bernadette Basuta Mirembe, Ingrid Hammermeister Nezu, Patricia Ndumbi, Julia Fitzner, Maria Almiron, Marcelo Vila, Richard Pebody, Aisling M. Vaughan, Joana M. Haussig, Luis Alves de Sousa, Okot Charles Lukoya, Olaniyi Felix Sanni, Pierre Nabeth, Jeremias Domingos Naiene, Masaya Kato, Tamano Matsui, Krutika Kuppalli, Peter Omondi Mala, Rosamund F. Lewis, Olivier le Polain de Waroux, Boris I. Pavlin, WHO Mpox surveillance and Analytics Team

**Affiliations:** Health Emergencies Programme, World Health Organization (WHO), Geneva, Switzerland (A. Hoxha, H. Laurenson-Schafer, N. Sklenovska, B.B. Mirembe, I.H. Nezu, P. Ndumbi, J. Fitzner, K. Kuppalli, P.O. Mala, R.F. Lewis, O. le Polain de Waroux, B.I. Pavlin);; CPC Analytics, Berlin, Germany (S.M. Kerr);; WHO Regional Office for the Americas, Washington, DC, USA (M. Almiron, M. Vila);; WHO Regional Office for Europe, Copenhagen, Denmark (R. Pebody, A.M. Vaughan);; European Centre for Disease Prevention and Control, Solna, Sweden (J.M. Haussig, L. Alves de Sousa);; WHO Regional Office for Africa, Brazzaville, Republic of the Congo (O.C. Lukoya, O.F. Sanni);; WHO Regional Office for the Eastern Mediterranean, Cairo, Egypt (P. Nabeth, J.D. Naiene);; WHO Regional Office for South-East Asia, Delhi, India (M. Kato);; WHO Regional Office for the Western Pacific, Manila, Philippines (T. Matsui)

**Keywords:** mpox, monkeypox, viruses, children, adolescents, outbreak, monkeypox virus

## Abstract

The 2022–2023 mpox outbreak predominantly affected adult men; 1.3% of reported cases were in children and adolescents <18 years of age. Analysis of global surveillance data showed 1 hospital intensive care unit admission and 0 deaths in that age group. Transmission routes and clinical manifestations varied across age subgroups.

Mpox is a zoonotic disease caused by monkeypox virus (MPXV) and previously found primarily in forested areas of Central and West Africa (*1*,*2*). In May 2022, a multicountry outbreak of mpox emerged; as of May 2023, there were >87,500 cases and 141 deaths reported from 111 World Health Organization (WHO) member countries (*3*). Globally 1.3% of reported cases during the outbreak have been in children and adolescents <18 years of age (*3*). During 1970–2021, mpox cases in Central Africa were predominately (54%–90%) reported in children (*4*–*6*), and children experienced more severe disease and adverse outcomes than adults (*6*,*7*).

After the first cases of mpox from countries without a history of the disease were reported to WHO in May 2022, a global surveillance system was established to collect aggregated data on probable and confirmed cases, as well as detailed case-based information on demographics, medical history, clinical manifestations, exposure factors, and testing (*8*). We describe epidemiologic and clinical characteristics of mpox in case-patients <18 years of age using surveillance data reported by all WHO regions during January 1, 2022–May 22, 2023 (https://www.who.int/publications/i/item/WHO-MPX-Surveillance-2022.4).

## The Study

**Figure 1:**
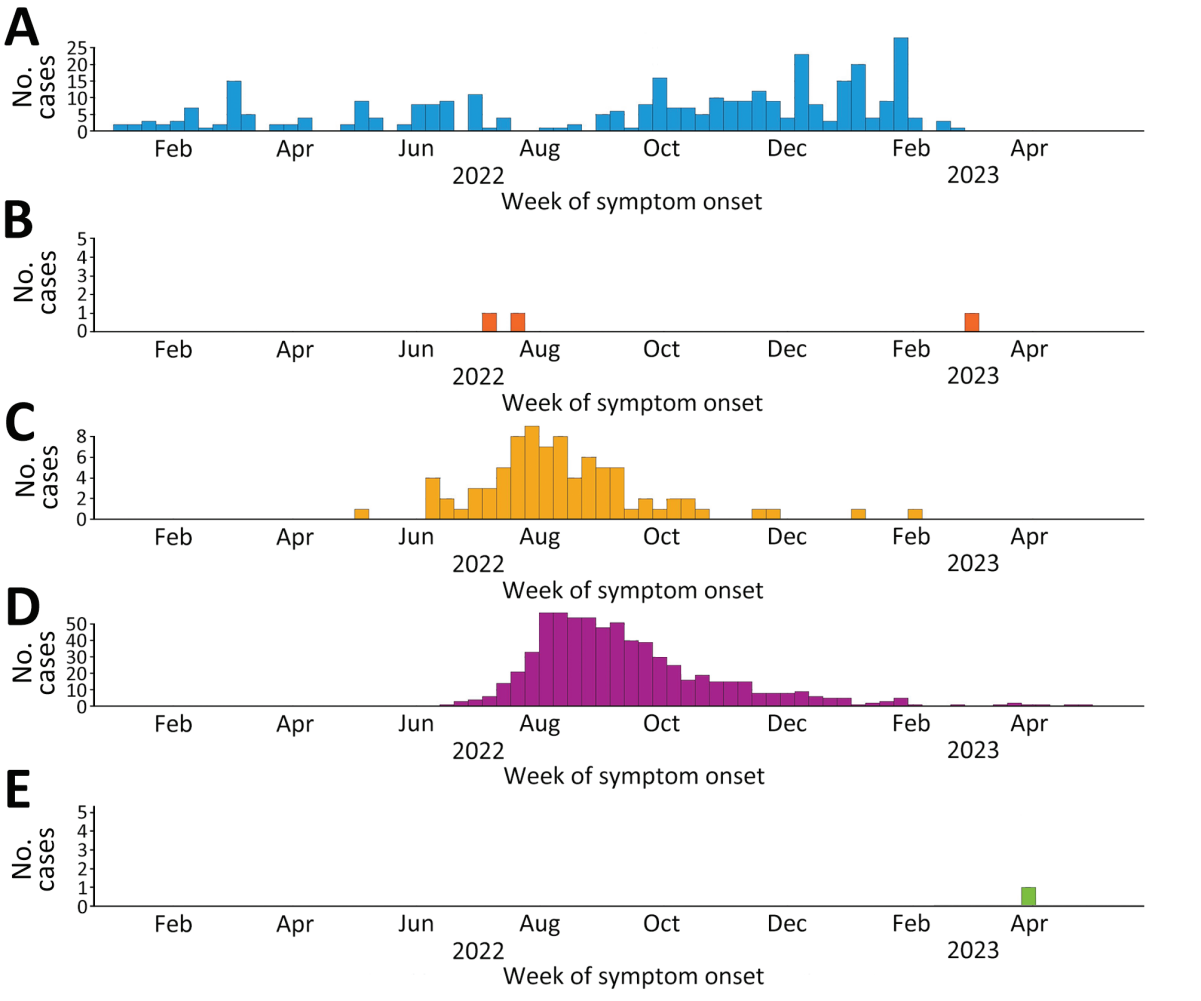
Epidemic curves of mpox cases among children and adolescents <18 years of age, grouped by World Health Organization regions, January 2022–May 2023. Dates represent the week of symptom onset, or the week of diagnosis or reporting if the date of symptom onset is unknown. A) African Region; B) Eastern Mediterranean Region; C) European Region; D) Region of the Americas; E) Western Pacific Region.

During the study period, 1.3% (1,118/84,614) of confirmed mpox cases globally were in patients <18 years of age. Most (61.8%, 691) were from the WHO Region of the Americas, followed by the African Region (30.3%, 339), the European Region (7.5%, 84), the Eastern Mediterranean Region, (<1%, 3), and the Western Pacific Region (<1%, 1) (Figure 1). No cases in patients <18 years of age were reported from the South-East Asia Region. Countries in the African Region reported mpox cases before the global outbreak (Figure 1), whereas reports of mpox cases in Europe and in the Americas began in May 2022 and peaked in July–August 2022. In Europe and the Americas, the epidemic curve of case-patients <18 years of age closely mimics the overall regional curves (*3*). The global percentage of case-patients <18 years of age has consistently remained low (0%–3%). By May 2023, overall case counts were low across all regions; the Americas, the Eastern Mediterranean Region, and the Western Pacific Region reported sporadic case-patients <18 years of age. Among the 1,102 case-patients <18 years with available information, 59.3% (654) were male and 40.7% (448) female (Appendix Figure). 

MPXV has 2 distinct clades, I and II (*9*). Virus clade information was not available for most cases and was assumed on the basis of the reporting country or subnational area and circulating clades in 2022, as reported by countries on GISAID (https://www.gisaid.org) or Nextstrain (https://nextstrain.org), in published literature, or to WHO. Of the 297 cases of mpox from countries reporting clade I, all age groups had a similar sex distribution (Appendix Figure, panel A). Of the 805 cases of mpox from countries reporting clade II, there were more male than female case-patients (269 vs. 104) among those 13–17 years of age. (For cases from Cameroon, 4 from eastern Cameroon are included in the clade I distribution, 1 from western Cameroon is included in the clade II distribution, and 1 for which we had no detailed geographic information was excluded.) Of case-patients with available hospitalization data, 47/335 (14.0%) were hospitalized and 1 was admitted to the intensive care unit; none was reported to have died.

Data on self-reported sexual behavior were limited for mpox case-patients <18 years of age. However, 37/166 (22.3%) of case-patients 13–17 years of age self-identified as men who have sex with men (MSM) (Table 1). Eleven cases involved persons living with HIV, 1 of whom was immunosuppressed. Another 6 case-patients reported immunosuppression, as defined by their care providers, that was caused by undisclosed medical conditions. Overall, 67/293 (22.9%) of cases with information provided had a stated epidemiologic link to a known mpox case.

**Table 1:** Main epidemiologic characteristics of mpox in children and adolescents reported globally, January 2022–May 2023*

Characteristic	No. (%) case-patients, by age group			
	0–4 y	5–12 y	13–17 y	Total
Total	328 (29.3)	353 (31.6)	437 (39.1)	1,118 (100)
Sex				
M	168 (51.2)	182 (51.6)	304 (69.6)	654 (58.5)
F	154 (47.0)	170 (48.2)	124 (28.4)	448 (40.1)
Unknown	6 (1.8)	1 (0.3)	9 (2.1)	16 (1.4)
Sexual behavior				
MSM	0	0	37 (8.5)	37 (3.3)
Non-MSM	0	43 (12.2)	119 (27.2)	162 (14.5)
Unknown	328 (100.0)	310 (87.8)	281 (64.3)	919 (82.2)
HIV status				
HIV+	0	2 (0.6)	9 (2.1)	11 (1.0)
HIV−	84 (25.6)	94 (26.6)	163 (37.3)	341 (30.5)
Unknown	244 (74.4)	257 (72.8)	265 (60.6)	766 (68.5)
Immunosuppressed				
Yes	1 (0.3)	2 (0.6)	4 (0.9)	7 (0.6)
No	116 (35.4)	119 (33.7)	176 (40.3)	411 (36.8)
Unknown	211 (64.3)	232 (65.7)	257 (58.8)	700 (62.6)
Known epidemiological link				
Yes	26 (7.9)	18 (5.1)	23 (5.3)	67 (6.0)
No	41 (12.5)	51 (14.4)	134 (30.7)	226 (20.2)
Unknown	261 (79.6)	284 (80.5)	280 (64.1)	825 (73.8)

*Percentages are calculated by column for each epidemiologic characteristic grouping. Unknown means that the information for the variable is missing. MSM, men who have sex with men.

**Figure 2:**
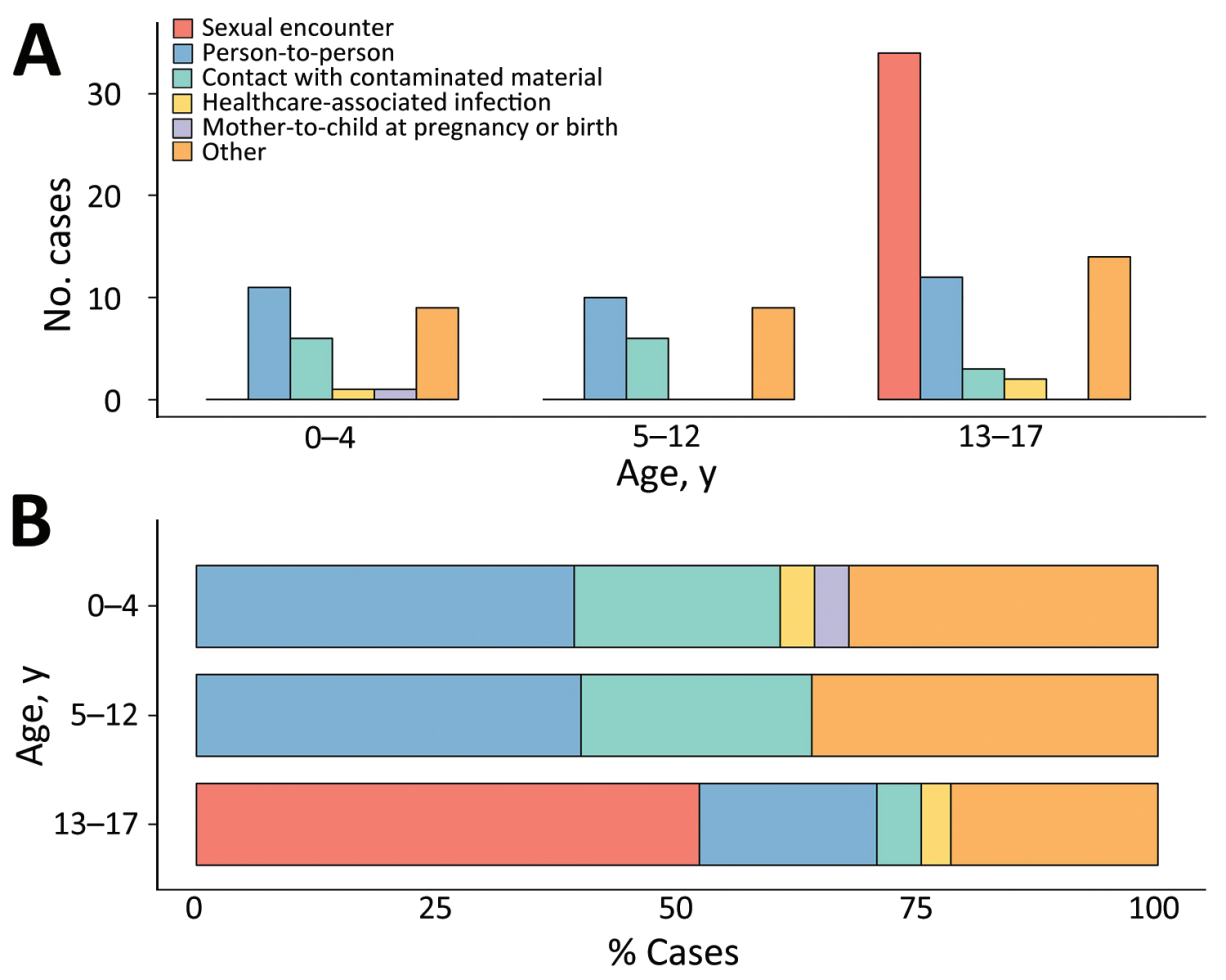
Mpox cases by transmission type by age group among children and adolescents <18 years of age, as reported globally to the World Health Organization, January 2022–May 2023. A) Absolute numbers; B) percentage among all cases with available information.

Case-patients 0–12 years of age were exposed to MPXV mainly through physical person-to-person contact (excluding sexual contact) or contact with fomites, whereas exposure through sexual encounter was exclusively reported by those 13–17 years of age (Figure 2). Among the older group, some of whom self-identified as MSM, sexual transmission may explain the unequal sex distribution of clade II cases (Appendix Figure, panel B). The type of transmission was reported as other in 32/118 (27.1%) of cases, without additional information.

Among cases with data, 472/542 (87.1%) experienced symptoms (Table 2). We excluded from analysis cases reported to be symptomatic but without any specific symptom. The presence of any rash, which consisted of >1 rash symptoms (on the body, oral, genital, or unknown location), was the predominant symptom for all age groups (325/542, 60%); genital rash was present in 54/229 (23.6%) of case-patients 13–17 years of age and 19/313 (6.1%) of those <13 years of age (p<0.00001). Among case-patients 13–17 years of age who reported being infected through sexual contact, genital rash was present in 15/34 (44.1%). Genital rash may be indicative of the transmission route of mpox but can also be present when transmission has not occurred through sexual contact. After rash, the most reported symptoms were fever (270/542, 49.8%) and headache (158/542, 29.2%). Lymphadenopathy has been reported as a common mpox symptom (*1*,*6*); it was reported in 60/542 (11.1%) of cases in our study.

**Table 2:** Main symptoms of mpox in children and adolescents reported globally among cases with symptom data, January 2022–May 2023*

Characteristic	No. (%) case-patients, by age group			
	0–4 y	5–12 y	13–17 y	Total
Total	156 (28.8)	157 (28.9)	229 (42.3)	542 (100)
Any symptoms				
Yes	124 (79.5)	132 (84.1)	216 (94.3)	472 (87.1)
No	32 (20.5)	25 (15.9)	13 (5.7)	70 (12.9)
Any rash				
Yes	77 (49.4)	84 (53.5)	164 (71.6)	325 (60.0)
No	79 (50.6)	73 (46.5)	65 (28.4)	217 (40.0)
Genital rash				
Yes	8 (5.1)	11 (7.0)	54 (23.6)	73 (13.5)
No	148 (94.9)	146 (93.0)	175 (76.4)	469 (86.5)
Fever				
Yes	66 (42.3)	73 (46.5)	131 (57.2)	270 (49.8)
No	90 (57.7)	84 (53.5)	98 (42.8)	272 (50.2)
Headache				
Yes	18 (11.5)	44 (28.0)	96 (41.9)	158 (29.2)
No	138 (88.5)	113 (72.0)	133 (58.1)	384 (70.8)
Any lymphadenopathy				
Yes	4 (2.6)	11 (7.0)	45 (19.7)	60 (11.1)
No	152 (97.4)	146 (93.0)	184 (80.3)	482 (88.9)

*Percentages are calculated by column for each symptom.

## Conclusions

During this outbreak, countries with high caseloads reported most of the case-patients <18 years of age, however, the percentage of pediatric and adolescent patients in those countries was lower than that for adults. The percentage of patients <18 years of age was lower than had been feared early in the outbreak, amid concerns that the epidemic could shift from primarily affecting MSM to a more generalized epidemic spread, including among school-age children. Epidemiologic and clinical characteristics were similar for the age groups 0–4 and 5–12 years, whereas case-patients 13–17 years of age, who are more likely to be sexually active, commonly reported MSM sexual behavior, exposure through sexual contact, and having more genital lesions.

Studies from the Netherlands (*10*), Spain (*11*), England (*12*), and the United States (*13*,*14*) have described MPXV infection among children and adolescents during this outbreak. Consistent with our findings, most cases in those studies reported no intensive care unit admissions or deaths; WHO is aware of the death of an infant from mpox clade I for which data are not available (*3*). Our results differ from historical reports of mpox in children, in which they have been described as at higher risk for adverse events and death related to disease (*15*). A previous study using the same surveillance data found higher odds of hospitalization for children <5 years of age than for those 15–45 years of age (*8*). The lower observed severity in children and adolescents in this outbreak than for previous outbreaks may be caused by a combination of increased ascertainment of mild cases, differing access to healthcare between settings, differing health status of the host populations, and lower virulence of clade IIb MPXV; clade I MPXV infection has been reported to be associated with higher severity than clade II (*1*).

Our study is based on surveillance data and likely underestimates the true number of case-patients <18 years of age, particularly in Africa (*8*). Disease severity may be underestimated because countries may not have updated case outcomes if hospitalization or death was delayed. Finally, data completeness varied among regions and countries; thus, our results may not be representative of each setting.

This study highlights the need for thorough epidemiologic investigation of mpox in children and adolescents. Clinicians should consider mpox as a possible diagnosis in these age groups when they have indicative symptoms, even with no known epidemiologic link to another case.

Appendix Additional information about mpox in children and adolescents during a multicountry outbreak, 2022–2023.
