# Supplementary material for: Mpox in Children and Adolescents during Multicountry Outbreak, 2022–2023
Source: Emerg Infect Dis. 2023 Oct;29(10):2125–9. doi: 10.3201/eid2910.230516 (PMC10521596; doi:10.3201/eid2910.230516)
Supplement: Appendix — Additional information about mpox in children and adolescents during a multicountry outbreak, 2022–2023. [file 23-0516-Techapp-s1.pdf]

Article DOI: http://doi.org/10.3201/eid2910.230516

EID cannot ensure accessibility for supplementary materials supplied by authors. Readers who have difficulty accessing supplementary content should contact the authors for assistance.

Mpox in Children and Adolescents during Multicountry Outbreak, 2022–2023

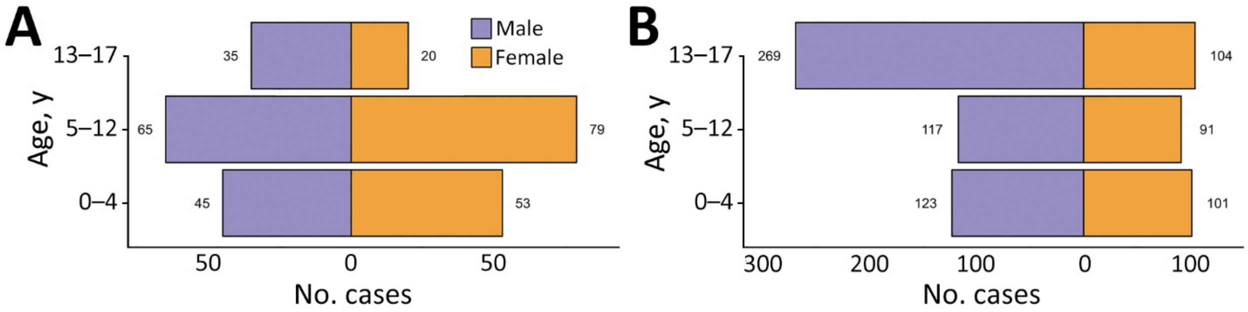

Appendix Figure. Age and sex distribution of mpox cases among children and adolescents reported globally to the World Health Organization, grouped according to the virus clade, January 2022–May 2023. A) Clade I. B) Clade II.
